# Sensitivity and Specificity of Qualitative Visual Field Tests for Screening Visual Hemifield Deficits in Right-Brain-Damaged Stroke Patients

**DOI:** 10.3390/brainsci14030235

**Published:** 2024-02-29

**Authors:** Maria De Luca, Fabrizio Zeri, Alessandro Matano, Concetta Di Lorenzo, Maria Paola Ciurli, Martina Mulas, Virginia Pollarini, Stefano Paolucci, Davide Nardo

**Affiliations:** 1 IRCCS Fondazione Santa Lucia, 00179 Rome, Italy; a.matano@hsantalucia.it (A.M.); t.dilorenzo@hsantalucia.it (C.D.L.); p.ciurli@hsantalucia.it (M.P.C.); s.paolucci@hsantalucia.it (S.P.); 2 Department of Materials Science, University of Milano-Bicocca, 20125 Milan, Italy; fabrizio.zeri@unimib.it; 3 Faculty of Medicine and Psychology, Sapienza University of Rome, 00185 Rome, Italy; 4 Department of Education, University of Roma Tre, 00185 Rome, Italy; davidenardo@gmail.com

**Keywords:** confrontation tests, hemianopia, quadrantanopia, visual field, right-brain stroke, sensitivity, specificity, kinetic boundary perimetry

## Abstract

A timely detection of visual hemifield deficits (VHFDs; hemianopias or quadrantanopias) is critical for both the diagnosis and treatment of stroke patients. The present study determined the sensitivity and specificity of four qualitative visual field tests, including face description, confrontation tests (finger wiggle), and kinetic boundary perimetry, to screen large and dense VHFDs in right-brain-damaged (RBD) stroke patients. Previously, the accuracy of qualitative visual field tests was examined in unselected samples of patients with heterogeneous aetiology, in which stroke patients represented a very small fraction. Building upon existing tests, we introduced some procedural ameliorations (incl. a novel procedure for kinetic boundary perimetry) and provided a scoresheet to facilitate the grading. The qualitative visual field tests’ outcome of 67 consecutive RBD stroke patients was compared with the standard automated perimetry (SAP; i.e., reference standard) outcome to calculate sensitivity and specificity, as well as positive and negative predictive values (PPV and NPV), both for each individual test and their combinations. The face description test scored the lowest sensitivity and NPV, while the kinetic boundary perimetry scored the highest. No test returned false positives. Combining the monocular static finger wiggle test (by quadrants) and the kinetic boundary perimetry returned the highest sensitivity and specificity, in line with previous studies, but with higher accuracy (100% sensitivity and specificity). These findings indicate that the combination of these two tests is a valid approach with RBD stroke patients, prompting referral for a formal visual field examination, and representing a quick, easy-to-perform, and inexpensive tool for improving their care and prognosis.

## 1. Introduction

When a stroke occurs in either the right or left hemisphere, it can lead to a range of consequences, from a contralesional visual field impairment to cognitive and/or motor deficits. A lateralised loss of visual sensory input is a negative prognostic factor, since it hampers both cognitive and motor rehabilitation. The prevalence of visual field losses in stroke patients ranges between 30% and 70%, with most studies agreeing on at least 50% of visual hemifield deficits (VHFDs) [1,2,3,4,5]. Among VHFDs in stroke patients, homonymous hemianopias are more frequent in those with a right-brain damage (RBD) than in those with a left one (e.g., [3,4]), and hemianopias are more frequent than quadrantanopias [2,3,4,5].

Large and dense visual field losses, such as hemianopia or quadrantanopia, can be quickly and conveniently screened by qualitative visual field tests, such as kinetic boundary perimetry and confrontation tests (e.g., finger wiggle, finger counting, or colour confrontation tests), before a formal (quantitative) examination is carried out by a standard automated perimetry (SAP) to make a diagnosis. The kinetic boundary perimetry [6], also known as peripheral fields test [7] is a quick and gross technique for assessing a patient’s visual field using a target (e.g., a red pin) that is moved to determine the boundary of the visual field. The test is based on the “from-unseen-to-seen” procedure, where the target is moved from the periphery towards the centre of the visual field, until the patient reports that the target is visible. The rationale of confrontation tests is that an examiner assesses the integrity of a patient’s visual field with respect to their own. The examination is performed monocularly by presenting targets (e.g., wiggling fingers) in the four quadrants of the visual field, and returns a gross measure of hemifield loss (e.g., hemianopia or quadrantanopia). Confrontation tests are classified into kinetic and static ones, depending on whether the examiner moves the targets from the periphery towards the centre of the visual field, or whether fixed positions are tested. Some qualitative visual field tests (i.e., face description test, kinetic boundary perimetry, colour comparison techniques, and central red field test) are commonly listed as confrontation tests, although strictly speaking they cannot be considered as such, since they are not based on a direct comparison with the examiner’s visual field (see [8]).

Confrontation tests and kinetic boundary perimetry are commonly used by ophthalmologists and optometrists to quickly screen deficits caused by pathologies such as glaucoma or intra-cranial tumours [8]. Confrontation tests are also included in the standard neurological examination as part of a comprehensive set of tests to screen visual loss with neurological aetiology [9]. Smaller central or paracentral deficits (such as small absolute or relative scotomas, scattered, or arcuate defects) can be ascertained only by a formal examination with a quantitative perimetry (SAP). However, qualitative testing based on kinetic boundary perimetry with central field analysis, which moves “from-seen-to-unseen”, may possibly detect some cases [10].

Despite their gross and non-standardised nature, qualitative visual field tests may help in detecting and screening gross deficits in stroke patients, thus representing a non-invasive, flexible, quick, and inexpensive tool to avoid delays in figuring out the clinical picture of a patient and defining a treatment plan. Treatments for visual field losses such as hemianopia or quadrantanopia following a stroke can be chosen among restorative, substitutive, and compensatory interventions (e.g., [11,12]). Restorative (or restitutive) interventions are based on the stimulation of the hemianopic hemifield, aiming to the recovery of the impaired visual field. Substitutive interventions are based on devices such as optical prisms. Compensatory interventions rely on the intact portion of the visual field and use visual scanning techniques. A recent systematic review and meta-analysis of randomised control trials identified five studies (totalling 206 patients with hemianopia), based on which it was concluded that interventions based on visual scanning or visual search (i.e., compensatory interventions) are effective treatments for hemianopia [13]. Of course, depending on a given hospital or rehabilitation unit’s facilities, not every stroke patient with a visual field deficit will have the opportunity to receive a timely intervention (e.g., [14]). Anyway, since neuroplasticity plays a fundamental role in the prognosis of hemianopia (e.g., [15]), and the prospects for improvement are strictly related to the timeliness of the intervention, a timely screening with qualitative tests or a diagnosis with an SAP is essential. Indeed, qualitative tests are often the only examination carried out during a patient’s hospitalisation [9].

Several studies have addressed the issue of sensitivity and specificity of qualitative visual field tests (including confrontation tests), and the issue of which test (or combination of tests) is the most effective in detecting visual field losses (e.g., [16,17,18,19,20,21,22]). These studies agree that the confrontation technique presents several limitations, and that the accuracy varies across tests. Notably, the *central field test to 5 mm red target* (deployed statically in various points within the central 20 deg. field) is the most sensitive for small deficits [19]. Additionally, the *static finger wiggle test* is more sensitive than the *finger counting test* [17]. Finally, any combination of tests that includes the *kinetic boundary testing with a red target* achieves the highest sensitivity in unselected samples of patients with a predominant percentage of anterior visual pathway lesions [17,19]. Based on such evidence, the use of qualitative tests has not been encouraged much in the past and, in case, it has been recommended to consider a combination of tests, rather than solely relying on individual tests, as combining tests enhances the probability of detecting a deficit, if present. However, it should be considered that the accuracy of these tests also depends on the type and density of the visual field loss, and therefore, on its aetiology. It is noteworthy that the patients examined in the above-mentioned studies were made up of unselected samples recruited from eye/ophthalmology clinics, with most patients suffering from pre-chiasmatic pathologies with heterogeneous aetiology (e.g., glaucoma, optic neuritis, ocular tumour, etc.), whereas—to the best of our knowledge—only a small fraction of stroke patients have been examined in studies addressing qualitative tests (ranging from n = 3 to n = 16 across studies). Therefore, currently in the literature there seems to be a sampling bias when assessing the sensitivity and specificity of these screening tests. Conversely, the present study was designed considering that the aetiology of a given disorder is essential when assessing the accuracy of qualitative tests, and hence we included only stroke patients.

As RBD stroke patients suffer from acquired brain injury, they may exhibit difficulties due to the co-occurrence of cognitive (e.g., attentional), primary sensory, and/or motor deficits. One of the most common attentional deficits is left unilateral spatial neglect (neglect, from here on). Its presence makes patients unaware of the contralesional side of space, even in the absence of a primary sensory loss. Neglect and VHFDs often co-occur, although they may exist independently from each other, as a double dissociation was demonstrated between sensory and attentional impairments [23]. The prevalence of visual field losses in stroke patients with neglect is about 50%, possibly underestimated [24,25,26,27,28]. Additionally, some neglect patients may exhibit visual extinction, in which the presence of a contralesional stimulus is detected in isolation, but remains undetected when presented concurrently with an ipsilesional stimulus (e.g., [29]). Therefore, in neurological and neuropsychological settings it is common practice to test extinction while administering confrontation tests, as it is the case in the present study.

The aim of the present study was to determine the sensitivity and specificity of four qualitative visual field tests to screen large and dense VHFDs in a large sample of RBD stroke patients. In doing so, we also introduced and described some procedural ameliorations with respect to existing procedures that keep into account RBD stroke patients’ potential attentional/motor problems and difficulties in performing the task. The four tests were: (i) a *face description* test (in monocular vision), as a simple task to start off the examination; (ii) a *static finger wiggle test* in binocular vision (from here on: *binocular static finger wiggle test*), with unilateral (single) and bilateral (double simultaneous) stimulation of the two hemifields (this test detects either hemianopia or extinction; cf. [30,31]); (iii) a *static finger wiggle test* in monocular vision (from here on: *monocular static finger wiggle test*) with unilateral (single) and bilateral (double simultaneous) stimulation of the upper and lower quadrants (this test detects either hemianopia or quadrantanopia, or extinction; cf. [8]); (iv) a *kinetic boundary perimetry* carried out using the red target of the Aston Perimetry Tool [10] (which is moved along arc trajectories), for which we developed a novel procedure (see Section 2 for details).

## 2. Materials and Methods

### 2.1. Participants

Seventy-three consecutive inpatients with acquired RBD (largely overlapping—75%—the sample described in [32]), referred for routine assessment following a stroke in the right-hemisphere, participated in the study. Inclusion criteria were as follows: age ≥ 18 years; a (haemorrhagic or ischaemic) lesion following a stroke in the right hemisphere (determined based on neuroimaging data). Exclusion criteria were: the presence of a lesion in the left hemisphere (determined based on neuroimaging data); the presence of severe cognitive deficits preventing the comprehension of instructions and/or the feasibility of the exam; a previous history of other neurological or psychiatric disease, or drug abuse; and the presence of severe visual deficits independent of stroke that would make the SAP unfeasible. The study was approved by the local Ethics Committee (CE/PROG.839 29-05-20). All patients gave their written informed consent to participate in the study.

To determine the sample size for the present screening study, a prevalence of 50% of VHFDs in stroke patients was considered [1,2,3,4,5], setting pre-determined null and alternative hypotheses to 50% and 80%, respectively, statistical power to 0.8, and significance to *p* < 0.05 [33]. The minimum number of patients required was 40, with 20 as the minimum sample size for a positive case. The final sample included 67 patients (see Table 1 for demographic and clinical data), given that two patients dropped out, two were excluded because the formal visual field assessment was unreliable (one for an excessive number of false positives, i.e., >15%; one for fixation losses, i.e., >33%), and two because it could not be performed (e.g., patients were either bedridden or semi-reclining in a wheelchair).

### 2.2. Formal Visual Field Assessment

A standard automated perimetry (SAP) was the reference standard used to evaluate the screening accuracy of qualitative visual field tests. The examination was carried out by a chartered orthoptist (C.D.L.) using a Humphrey Field Analyzer 3 (HFA; Zeiss Meditech). The SITA Faster strategy with stimulus size III was used to test the central 24-2 threshold separately for the left and right eye. The possible outcomes were: left homonymous hemianopia; left homonymous (superior or inferior) quadrantanopia; incongruous deficits, scotomas or scattered non-lateralized losses; or absent deficit. Patients were excluded from continuing to participate in the study based on the SAP reliability indexes (i.e., fixation losses, false positive, and false negative responses). No patient presented with visual impairments that prevented the assessment with the SAP.

### 2.3. Qualitative Visual Field Assessment

#### 2.3.1. General Procedure

The following testing procedure was validated by both a senior neurologist (S.P.) and a senior optometrist and researcher in vision science (F.Z.). All tests were performed in a well-lit room, with the patient’s eye(s) facing a homogeneous opaque light grey background. The same room was used for all patients. An adjustable seat for the examiner was part of the examination setting, to align the examiner’s eyes at the same height as the patient’s. Eyeglasses were removed in order not to obstruct the peripheral vision (all qualitative tests’ targets can be seen without any refractive correction). The tests were administered and interpreted by a senior researcher in neuropsychology and vision science (M.D.L.) blind to the initial diagnosis, lesion site, neuropsychological and formal visual field assessment outcomes. When testing was in monocular vision, the non-tested eye was occluded with an ophthalmic eye patch.

The *face description test* and *kinetic boundary perimetry* are monocular tests not based on confrontation (see [8]). Tests based on an actual confrontation are the *binocular static finger wiggle test* (to assess the visual field in the two hemifields, and the possible presence of visual extinction to simultaneous bilateral stimulation; see [30]), and the *monocular static finger wiggle test* (to assess the visual field in the four quadrants together with extinction, e.g., [8]). In both finger wiggle tests, not reporting the left stimuli when they are presented in isolation indicates a VHFD. Conversely, reporting the left stimuli when presented in isolation, but not reporting them when presented concurrently with the right ones (i.e., during simultaneous bilateral stimulation) indicates extinction. In both finger wiggle tests, we added some catch trials (that is, wiggling no finger). This was done for two reasons: (i) to check response reliability; and (ii) to avoid that a patient got anxious in the event of several missed targets in a row on the left side (cf. Discussion). The instructions about the four stimulus conditions of the finger wiggle tests (i.e., right, left, bilateral, or none) were given before testing, and repeated whenever necessary. Except for the *face description test* (that was always presented first, as it is an easy test to start off), the order of the other tests was randomized across patients. Maintenance of fixation was carefully checked for all patients. In the *finger wiggle* tests and *kinetic boundary perimetry*, any response provided following a loss of central fixation was discarded, and the trial was repeated (in random order) later. Importantly, RBD stroke patients typically present with attentional and/or motor deficits, and many of them appear to be afraid to stare into the examiner’s eye(s). Indeed, it has been shown that directly staring at a human eye may negatively influence contralesional performance in RBD stroke patients [34]. Therefore, a red dot patch (0.5 cm diameter) was placed onto the examiner’s tip of the nose to serve as a fixation point. Finally, patients’ responses were recorded on a specific scoresheet (see Scoresheet in the Supplementary Materials) by filling the scoring tables in, and mapping the VHFD onto the scoring grids. The examination was completed in a single session lasting approx. 20–40 min, depending on the patient. For all tests, the main outcome was binary, i.e., defined as a present or absent VHFD. The outcome for extinction was also reported either as present or absent. In addition, if a VHFD was present, then the patient’s responses mapped onto the scoring grids of the *monocular static finger wiggle test* or the *kinetic boundary perimetry* were used to further characterise the visual field loss (e.g., as hemianopia or inferior/superior quadrantanopia).

#### 2.3.2. Face Description Test

**Figure 1:**
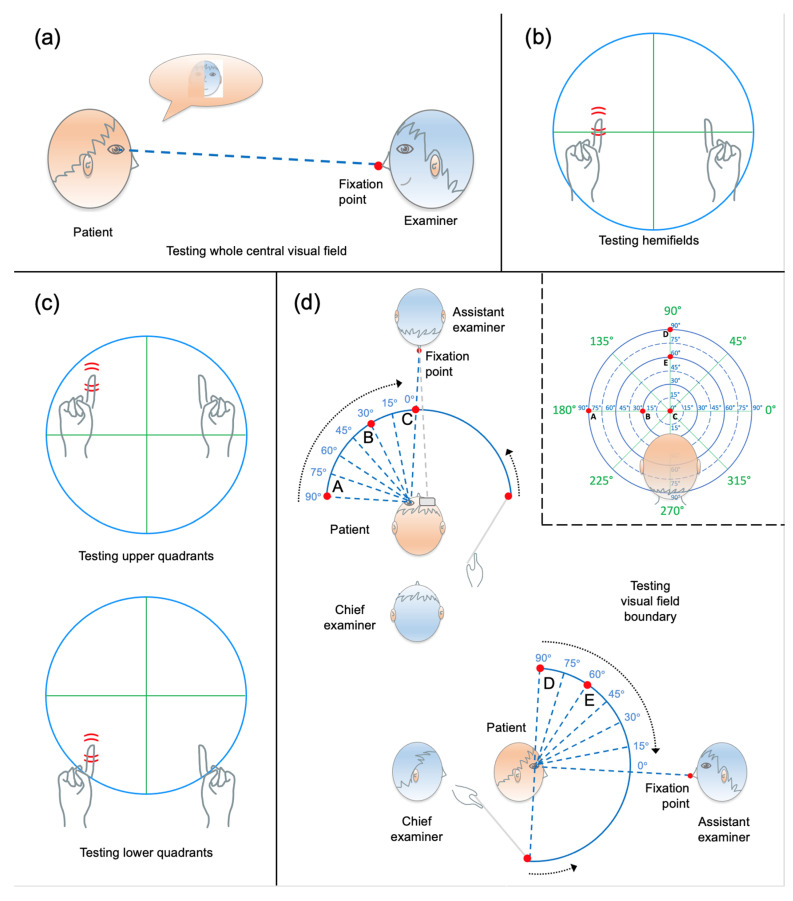
Sketch of the four qualitative visual field tests for illustrative purposes. (**a**) Face description test (performed separately for the right and left eye). (**b**) Binocular finger wiggle test. The panel shows the wiggling finger(s) at the height of the horizontal meridian, targeting the left and right hemifields from the patient’s perspective (who watches with both eyes open). (**c**) Monocular finger wiggle test. For either eye, the top panel shows the wiggling finger targeting the upper left quadrant from the patient’s perspective, while the bottom panel shows the wiggling finger targeting the lower left quadrant. Both the upper and lower quadrants are assessed in monocular vision. (**d**) Kinetic boundary perimetry. The test is performed separately for the right and left eye. Left panel—Bird’s-eye view of the patient’s position and visual field (blue arc). The red points (A, B, and C) illustrate exemplary positions of the red target (90°, 30°, and 0°, respectively; in blue) while testing a patient along the horizontal left meridian (180°; in green). Bottom panel—Side view of the patient’s position and visual field (blue arc). The red points (D and E) represent exemplary positions of the red target (90° and 60°, respectively; in blue) while testing a patient along the vertical upper meridian (90°; in green). Right panel—Projections of the target positions onto the scoring grid. The red points on the grid correspond to the red target positions shown in the left panel (points A, B, and C for the horizontal meridian 180°; in green), and in the bottom panel (points D and E for the vertical meridian 90°; in green). The patient’s head is shown to get a sense of the chief examiner’s perspective.

The right and left eyes were examined separately. The examiner sat in front of the patient at an eye-to-eye distance of approx. 65 cm (Figure 1a). The patient was then instructed to fixate on the red dot patch and indicate whether they could see the face of the examiner clearly, or whether any part of it appeared blurred or missing. In ordinary conditions (i.e., with a patient who is alert, well-oriented, cooperative, etc.), this test takes about 2 min to be administered (incl. swapping the eye patch), but its duration can be longer otherwise, as often patients spontaneously list all parts and details of a face.

#### 2.3.3. Binocular Static Finger Wiggle Test

This test examines the right and left peripheral visual hemifields at the height of the horizontal meridian (i.e., the upper and lower quadrants were not examined separately; see Discussion for potential pitfalls with this approach). The eye-to-eye distance between the examiner and the patient was approximately 65 cm. The examiner widened their arms and placed the fists (with the index fingers pointing upwards) in the frontal plane, halfway between themselves and the patient. The wiggling tips of the index fingers (i.e., the targets) were located at the height of the horizontal meridian of the visual field (Figure 1b). The examiner made sure to see the targets while looking at the patient (each target was approx. 50 deg. of eccentricity relative to fixation). Both index fingers were always present on either side with respect to the vertical meridian. The finger could wiggle either on one side (right or left), or bilaterally. This latter condition assessed the possible presence of visual extinction. The patient was also told that sometimes the examiner would not wiggle any finger at all when asking for a response (catch trials). To start the test, the examiner asked the patient to keep their gaze steady on the fixation point and report on which side the finger(s) moved (right, left, both, or none). The patient was instructed to respond based on their perspective, using a few training trials before the actual examination began. The first target was presented in the right visual hemifield of the patient, to start off with a stimulus likely to be detected. Then, all four conditions were randomly tested at least five times each (i.e., for a total of 20 trials). In ordinary conditions, this test takes about 2–4 min. to be administered.

#### 2.3.4. Monocular Static Finger Wiggle Test

This test examined the peripheral visual field in the four quadrants (upper left and right, and lower left and right). The eye-to-eye distance between the examiner and the patient was approximately 65 cm. When the patient’s right eye was examined, the examiner closed their own left eye, and vice versa. Then, as in the binocular test, the examiner placed the index fingers and used them as targets (approx. 30 deg. of eccentricity relative to fixation, either in the upper or lower quadrants; see Figure 1c). Both index fingers were always present, and could wiggle either on one side, bilaterally, or not wiggle at all (catch trials). The examiner asked the patient to maintain their gaze steady on the fixation point, and report on which side the finger(s) moved. The test was carried out randomly alternating the four conditions, until they were tested at least three times each, separately in the upper and lower quadrants (totalling 48 trials). In the event of a visual loss detection, additional positions within a quadrant were examined to refine its extension (e.g., [19]). In ordinary conditions, this test takes about 3–7 min to be administered.

#### 2.3.5. Kinetic Boundary Perimetry

This test was used to examine the boundary of the visual field (peripheral isopter). The right and left eyes were examined separately. Differently from the setting adopted by [7], two examiners (a chief examiner and an assistant examiner) cooperated to carry out the test, and the chief examiner was assigned a rear position (see Figure 1d), in order not to affect the patient’s performance by letting them unintentionally predict the starting position of the target based on a movement of the examiner’s arm. The assistant examiner (with the red dot patch on the tip of the nose) sat in front of the patient, to monitor the maintenance of fixation, and warn the chief examiner in case of invalid trials due to a displacement of the patient’s eyes. The chief examiner stood behind the patient, holding and manoeuvring the rod (Aston Perimetry Tool [10]). The tool is a 33 cm long, thin transparent stick, with a 15 mm red sphere on one end and a 5 mm white sphere on the other end. The red sphere was used as a target. The chief examiner held the tool, keeping the white sphere tangential to the head of the patient, and moved the rod so that the red target made an arc trajectory from the periphery to the centre of the patient’s visual field (see left and bottom panels in Figure 1d) along 8 radial meridians (0°, 45°, 90°, 135°, 180°, 225°, 270° and 315°; see Figure 1d, right panel). The arc was centred on the patient’s eye at a distance of about 33 cm. The speed of the target was approx. 5–7 deg./s. The patient was required to look at the fixation point, and report as soon as the target appeared in their visual field. Each trajectory was examined three times (or more in case of unreliable trials, or loss of central fixation), and the point of the visual field where the patient detected the target was recorded in the Scoresheet by the chief examiner. The test was performed using the “from-unseen-to-seen” procedure; therefore, the visual field was not further inspected for possible scotomas, even if the tool would allow such a testing (see [10]). In the event of a visual loss detection, additional positions between meridians were examined. In ordinary conditions, this test takes about 5–10 min to be administered.

### 2.4. Data Analysis

Descriptive statistics for demographic and clinical data were computed and reported in Table 1. The qualitative tests binary outcomes (that is, the detections of the presence or absence of a VHFD) were compared to the SAP outcomes to determine the number of true/false positive/negative cases (see individual data reported in the Supplementary Table S1). On this basis, sensitivity and specificity, as well as positive and negative predictive values (PPV and NPV) and confidence intervals (95% CI) were computed for each individual qualitative visual field test (see Table 2a). PPV and NPV were computed considering a 50% prevalence [35]. Sensitivity and specificity were also determined for the 11 combinations of the 4 tests (see Table 2b). For each combination (whether a 2-test, 3-test, or 4-test combination), a true positive case was returned if either test detected a VHFD, and a true negative if neither test detected a VHFD. Sensitivity and specificity of the qualitative visual field tests were determined for both the overall sample (see Section 3.3 and Table 2) and for a subsample of 44 patients with neglect (see Supplementary Methods and Supplementary Results). In addition to the qualitative visual field tests binary outcome, we also characterised the type of deficit detected by the *monocular static finger wiggle test* and *kinetic boundary perimetry*, to consider the exact nature of the VHFD detected (e.g., hemianopia, or superior or inferior quadrantanopia). This allowed us to compute the number of cases in which the type of VHFD detected by each of these two qualitative tests matched the type of VHFD diagnosed by the SAP. The results for extinction are reported by individuals in Supplementary Table S1, without any further analysis.

## 3. Results

### 3.1. Participants

Results by group and individual patients are reported in Table 1 and Supplementary Table S1, respectively. A total of 23 patients presented with a VHFD: 18 had left homonymous hemianopia, 4 left homonymous inferior quadrantanopia, and 1 left homonymous superior quadrantanopia. The remaining 44 did not present with any VHFDs.

As part of the routine neuropsychological assessment, RBD stroke patients underwent a battery for neglect. The comprehensive list of tests can be found in the Supplementary Methods. The cut-offs used to classify participants into patients with neglect (N+) and without neglect (N−) can be found in [32]. As a result of the neglect battery, there were 44 N+ and 23 N− patients (see Table 1).

**Table 2:** Sensitivity, specificity, PPV and NPV (incl. 95% CI) for the qualitative visual field assessment are reported for the whole sample of right-brain-damaged (RBD) stroke patients (n = 67). Results are reported for (a) individual four tests, and (b) their combinations. Sensitivity, specificity, PPV, and NPV values are based on true/false positive/negative cases (cf. Supplementary Table S1) determined by comparing the qualitative visual field tests binary outcome (i.e., presence or absence of a deficit) with the SAP outcome. The best combination (see Discussion) is highlighted in italic text.

	RBD Stroke Patients (n = 67)			
(a) Individual Tests	Sensitivity (%)	Specificity (%)	PPV (%)	NPV (%)
Face description	13.0 (−0.7–26.8)	100 (100–100)	100 (100–100)	53.5 (41.3–65.7)
Binocular static finger wiggle	60.9 (40.9–80.8)	100 (100–100)	100 (100–100)	71.9 (59.8–84.0)
Monocular static finger wiggle	87.0 (73.2–100)	100 (100–100)	100 (100–100)	88.5 (79.3–97.6)
Kinetic boundary perimetry	91.3 (79.8–100)	100 (100–100)	100 (100–100)	92.0 (84.2–99.8)
**(b) Combinations of tests**				
**2-test**				
Face description + Binocular static finger wiggle	60.9 (40.9–80.8)	100	100	71.9 (59.8–84.0)
Face description + Monocular static finger wiggle	87.0 (73.2–100)	100	100	88.5 (79.3–97.6)
Binocular static finger wiggle + Monocular static finger wiggle	87.0 (73.2–100)	100	100	88.5 (79.3–97.6)
Face description + Kinetic boundary perimetry	95.7 (87.3–100)	100	100	95.8 (90.0–100)
Binocular static finger wiggle + Kinetic boundary perimetry	100 (100–100)	100	100	100
*Monocular static finger wiggle* + *Kinetic boundary perimetry*	*100 (100–100)*	*100*	*100*	*100*
**3-test**				
Face description + Binocular static finger wiggle + Monocular static finger wiggle	87.0 (73.2–100)	100	100	88.5 (79.3–97.6)
Face description + Binocular static finger wiggle + Kinetic boundary perimetry	100 (100–100)	100	100	100
Face description + Monocular static finger wiggle + Kinetic boundary perimetry	100 (100–100)	100	100	100
Binocular static finger wiggle + Monocular static finger wiggle + Kinetic boundary perimetry	100 (100–100)	100	100	100
**4-test**				
Face description + Binocular static finger wiggle + Monocular static finger wiggle + Kinetic boundary perimetry	100 (100–100)	100	100	100

### 3.2. Qualitative Visual Field Assessment

#### 3.2.1. Face Description Test

Three patients showed the presence of a deficit on this test. When compared to the SAP outcome, these cases represented the only true positive cases (all with hemianopia). There were also 20 false negatives (15 with hemianopia, 4 inferior quadrantanopias, and 1 superior quadrantanopia), and the remaining 44 were all true negatives.

#### 3.2.2. Binocular Static Finger Wiggle Test

The 14 patients who showed deficits on this test were all true positive cases when compared with the SAP outcome (13 with hemianopia and 1 with superior quadrantanopia), while there were 9 false negatives (5 with hemianopia, and 4 with inferior quadrantanopia), 44 true negatives, and no false positives. This test detected 12 patients showing extinction.

#### 3.2.3. Monocular Static Finger Wiggle Test

A total of 20 patients showed a deficit on this test. All of them were true positive cases: 15 were diagnosed with hemianopia, 4 inferior quadrantanopia, and 1 superior quadrantanopia using the SAP. There were 44 true negatives, and the remaining 3 patients were false negatives, all diagnosed with hemianopia using the SAP. Among the 20 true positive cases, the characterisation of the type of VHFD detected by this qualitative test (i.e., identifying the detected VHFD as hemianopia or quadrantanopia) resulted in 17 exact correspondences with the type of SAP outcome (12 hemianopias, 4 inferior and 1 superior quadrantanopias). The remaining three cases were three hemianopias, for which the confrontation test detected one inferior quadrantanopia, one superior quadrantanopia, and one incongruous deficit (hemianopia with the right eye, superior quadrantanopia with the left one). This test detected 18 patients showing extinction; however, they were not the same patients as in the monocular test (see Supplementary Table S1). The overlap between the two *finger wiggle* tests (i.e., patients showing extinction in both tests) was 11.

#### 3.2.4. Kinetic Boundary Perimetry

A total of 21 patients showed a deficit on this test. All of them were true positive cases: 16 were diagnosed with hemianopia, 4 with inferior quadrantanopia, and 1 with superior quadrantanopia using the SAP. There were 44 true negatives, and the remaining 2 patients were false negatives (both diagnosed with hemianopia using the SAP). Among the 21 true positive cases, the characterisation of the type of deficits detected with this gross perimetry returned 16 exact correspondences with the type of SAP outcome (11 hemianopias, 4 inferior quadrantanopias, and 1 superior quadrantanopia). The remaining five cases were five hemianopias, for which the gross perimetry detected three inferior quadrantanopias, and two cases with an incongruous deficit (one case showing hemianopia with the right eye and superior quadrantanopia with the left one, and one showing hemianopia with the right eye and inferior quadrantanopia with the left one).

### 3.3. Sensitivity and Specificity of the Qualitative Visual Field Assessment

**Figure 2:**
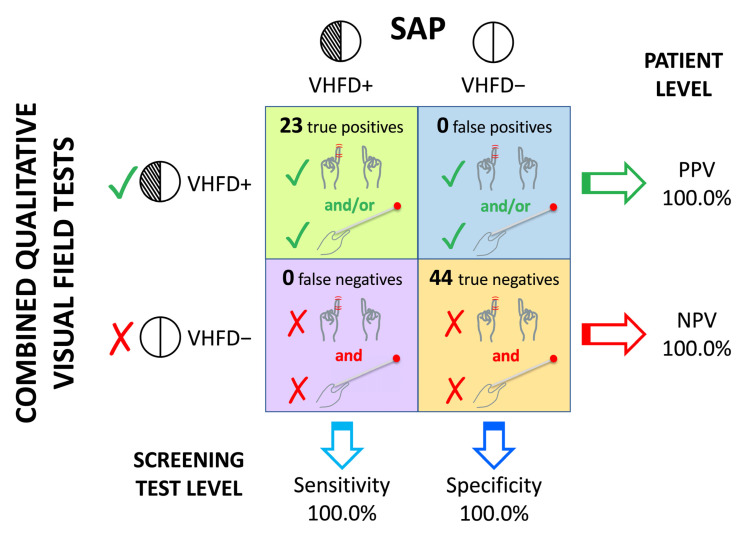
Sensitivity, specificity, PPV, and NPV for the combination of the *Monocular finger wiggle test* with the *Kinetic boundary perimetry* in the whole sample. The combination of these two qualitative visual field tests (sketched on the left) was used to screen visual hemifield deficits (VHFDs) in right-brain-damaged (RBD) stroke patients. When either screening test detected a deficit (green tick marks), the outcome was “presence of a VHFD” (VHFD+). When neither detected a deficit (red crosses), the outcome was “absence of a VHFD” (VHFD−). The screening outcome (VHFD+ or VHFD− by rows) was compared with the standard automated perimetry (SAP; reference standard) outcome (VHFD+ and VHFD− by columns) to determine the number of true/false positive/negative cases (shown in the four boxes), on which the four metrics of screening accuracy (sensitivity, specificity, PPV, and NPV) were computed. Sensitivity and specificity pertain to the screening tests, and represent the probability that their outcome corresponds to the reference standard outcome. Positive and negative predictive values (PPV and NPV) pertain to the patient level, and refer to the clinical performance of a screening test (i.e., the likelihood that a patient truly affected is screened as affected, and that a truly unaffected patient is screened as unaffected, respectively; e.g., [36]). The combination of these qualitative visual field tests achieved values equal to 100% for all the four metrics.

The sensitivity, specificity, PPV, NPV and 95% CI are presented in Table 2 for the individual tests, as well as their combinations. The best individual test was the *kinetic boundary perimetry* (sensitivity = 91.3%), followed by the *monocular finger wiggle test* (sensitivity = 87.0%). When the *kinetic boundary perimetry* was combined with any of the other tests, the figure of true positive cases improved, reaching a sensitivity of 100% for some combinations (see Figure 2). All combinations including the *kinetic boundary perimetry* (except the combination “*Face description* + *Kinetic boundary perimetry*”) returned the same sensitivity. Since N+ patients contributed with most VHFD cases (see Table 1), for further information, the sensitivity, specificity, PPV, NPV and 95% CI were also determined separately for this subsample of patients, and the results presented and discussed in the Supplementary Materials. The overall pattern of results in N+ (see Supplementary Table S3), overlapped with the results in the total sample presented in Table 2.

### 3.4. Characterisation of the Type of VHFD

The characterisation of the type of deficit showed that, individually, the *monocular static finger wiggle test* and the *kinetic boundary perimetry* correctly characterised the type of 17 and 16 VHFDs, respectively (73.9% and 69.6%), that is, all quadrantic deficits (n = 5) and more than half of the hemianopias (n = 12 and n = 11 out of 18 hemianopic deficits, respectively). However, the *monocular static finger wiggle test* did not match three hemianopias (it identified two different, and one incongruous deficit), while it returned no deficit in the remaining three cases with hemianopia (false negatives). The *kinetic boundary perimetry* did not match five hemianopias (it identified three different deficits, and two incongruous deficits), while it returned no deficit in the remaining two cases (false negatives; see Supplementary Table S1). Sensitivity and specificity were not computed for such characterisations, since the incongruous VHFD detected by the qualitative test(s) prevented us from classifying them as true/false positive/negative cases when compared to the deficit diagnosed by the SAP (e.g., the qualitative test detected an inferior quadrantanopia in the left eye, and a hemianopia in the right eye in a patient with hemianopia).

## 4. Discussion

Qualitative visual field tests may offer a quick, simple, and inexpensive means to detect possible visual field deficits before a formal test such as an SAP can be carried out. Oftentimes, the confrontation test included in the neurological examination is the only possible visual field assessment for stroke patients, when a formal computerized examination is unavailable or unfeasible [9,37]. This has implications for the diagnosis, prognosis, and treatment. The choice of adopting qualitative tests instead of a formal one is not based on tests duration, but rather on the availability of an SAP device as a facility at the clinical service/unit. An SAP typically requires 10–15 min per eye to return a formal diagnosis (totalling a maximum of about 30 min), while the set of qualitative tests adopted in the present study takes about 20–40 min for screening purposes (and about 10–20 min. when only the two most reliable tests are used).

From a practitioner’s perspective, if qualitative visual field tests are the only available tools to detect a suspected visual field deficit, then it is essential to make use of instruments tested in populations with the same characteristics (including aetiology) as the practitioner’s patients. For instance, the typical population examined in a neuro-ophthalmological clinic has a higher prevalence of visual field deficits than that typically examined in a neurological clinic (e.g., [38]). However, most of these deficits are due to pre-chiasmatic lesions, to which qualitative tests are not sensitive. Overall, it is important for the practitioner to consider that: (i) the sensitivity varies depending on the aetiology of the disorder; (ii) these tests are more sensitive to large and dense deficits; and (iii) different qualitative visual field tests return different outcomes due to their different sensitivity.

Keeping in mind the above-mentioned considerations, the present study is unique, in that it determines the sensitivity and specificity of qualitative visual field tests selectively in stroke patients, whereas previous studies did not distinguish among different aetiologies. In addition, we introduce a number of methodological ameliorations tailored to these specific patients.

To the best of our knowledge, the literature investigating the accuracy of qualitative tests is based on unselected samples of patients showing deficits caused by heterogeneous aetiologies that do not include stroke, or in which the number of stroke patients was not reported, or represented a negligible percentage (e.g., [16,17,18,19,20,21,22]). These studies showed that more than 20% of visual field deficits went undetected even when using the best individual test, or combinations of tests (e.g., 44% undetected by kinetic boundary [19]). In these studies, the sensitivity was higher for severe losses, and for retro-chiasmatic lesions with respect to pre-chiasmatic ones. However, the sensitivity for retro-chiasmatic lesions did not reach 80% (due to heterogeneous sampling, including chiasmatic lesions and retro-chiasmatic tumours along with a minority of stroke cases), while tests such as face description and finger counting tests showed low sensitivity even in cases of severe loss (e.g., 56% and 49%, respectively [17]). Only one study [21] reported 90% sensitivity for the monocular static finger wiggle test in 10 patients (with unknown aetiology) diagnosed with hemianopia. Therefore, the use of qualitative tests has not been encouraged in previous studies, whereas formal examinations with an SAP were strongly recommended.

The present study computed the sensitivity and specificity of qualitative visual field tests (including confrontation tests) in RBD stroke patients to provide information about the screening accuracy of qualitative tests in these patients. The present study also introduced procedural ameliorations and presented results showing that these tests can be considered valuable resources for screening large and dense visual field losses, and accelerate the diagnostic process (e.g., immediately refer a patient for an SAP). Some existing procedures were modified by adding or changing elements to address the potential attentional/motor difficulties of RBD stroke patients. Overall, the results indicate that selecting a specific combination of tests yields very high levels of accuracy (cf. [17]).

The four individual tests returned varying degrees of sensitivity (ranging from 13.0 to 91.3%, see Table 2a) and corresponding NPV (ranging from 53.5 to 92.0%), while specificity (and PPV) was maximum (100%) for all tests (see Table 2a), since there were no false positive cases. The sensitivity of individual tests in the present study was slightly higher than that reported in previous studies on unselected samples of patients, while the specificity was 100%, consistent with most studies in the literature (e.g., [17,19]). In the present study, the *kinetic boundary perimetry* was the test with the best sensitivity (91.3%), immediately followed by the *monocular static finger wiggle test* (87.0%). It is noteworthy that, not only these two tests were sensitive to the presence of a VHFD, but they could also accurately characterise the type of deficit, as shown by the percentages reported in Section 3.4.

Beyond the sensitivity and specificity of the qualitative tests, it is important to consider the PPV and NPV, since they pertain to the outcome of a patient’s screening. Any individual test or combination of tests with a high PPV (i.e., >90%) implies that if a test detects a visual field loss of any type, a visual deficit is likely to be present in a given patient. Regarding the NPV, high values indicate that, if a visual field loss is not detected, then that patient is likely not affected. Our results confirmed that the *kinetic boundary perimetry* is the best test, with 100% and 92% PPV and NPV, respectively. NPV achieved 100% only when this test was combined with the *monocular static finger wiggle test* (see below).

The sensitivity and NPV values were different across the four qualitative tests, because their procedures encompass various targets and task demands. These discrepancies underscore the importance of not relying only on a single test. Previous studies on unselected samples of patients determined the advantage of using combinations of tests and recommended their use (e.g., [17,19]). It has been shown that the kinetic red target combined with the static finger wiggle test achieve the best sensitivity (78.3%, higher than any single test) in a heterogeneous sample with 9.8% stroke patients [17]. Consistently with previous studies, our results showed that combining two or more qualitative tests achieve higher sensitivities (up to 100%) than a single test, and confirmed that the most sensitive combinations include the *kinetic boundary perimetry* (see Table 2b). However, the combination of the *kinetic boundary perimetry* and the *monocular static finger wiggle test* should be preferred to others (the sensitivity and NPV being equal), as such combination returns the most accurate result using only two tests, both of which are characterised by individual sensitivities higher than those of other individual tests. In fact, even if the combination of the *binocular finger wiggle test* and the *kinetic boundary perimetry* returns the same sensitivity and specificity as the combination of the *monocular finger wiggle test* and the *kinetic boundary perimetry*, the latter combination should be preferred. This is because, considered as individual tests (cf. Table 2a), the *monocular finger wiggle test* shows greater sensitivity (87.0%) as compared to the *binocular finger wiggle test* (60.9%). In addition, the latter is not particularly appropriate for stroke patients, given the risk of missing quadrantic deficits (cf. Section 4.1.4).

To address the issue of false negative cases affecting sensitivity and NPV, it should be considered that the sensitivity of qualitative tests may depend upon stimulus salience. Unlike the SAP, qualitative tests (like the ones used in the present study) make use of supra-threshold stimuli as targets (e.g., wiggling fingers or a red moving target), which are more salient than (and do not disappear as quickly as) the threshold- or near-threshold stimuli used in the SAP (i.e., small static white lights on a white background). Working with supra-threshold targets is more likely to result in false negatives because the blind portion of the visual field could still be sensitive to stimuli such as the high contrast moving objects used in the *static finger wiggle test* and *kinetic boundary perimetry*. In fact, despite appearing as dense visual losses in the SAP, hemianopias and quadrantanopias do not represent “all-or-none” deficits. Indeed, functional MRI studies in patients with quadrantanopia or hemianopia (e.g., [39,40]), showed visual cortical activity even in the absence of conscious stimulus detection (i.e., blindsight). Therefore, qualitative tests may not detect some hemianopias and quadrantanopias, even if they appear as complete deficits on the SAP. In addition, it should be considered that qualitative tests are not sensitive to scotomas or scattered deficits, unless the red target in the kinetic boundary perimetry is used under the “from-seen-to-unseen” procedure (that is, asking patients to report when the target disappears after having seen it). However, this procedure was not adopted in the present study, since it would have substantially increased testing time.

Finally, the present study introduced some changes to existing procedures previously adopted, in order to prevent and manage potential difficulties specifically arising with stroke patients, but that can be profitably adopted also with other types of patients. In fact, stroke patients may sometimes be non-compliant with instructions, even if they intend to be. For instance, they are typically concerned about the change in their health condition due to the stroke, and are afraid of failing tests. Therefore, while performing the tasks, they may inadvertently adopt behaviours that are functional in everyday life, but are undesirable in the testing context. Oftentimes, in an attempt to demonstrate their ability or to “please” the examiner with correct responses, they may shift their gaze looking for a target, or anticipate the position of a target cued by the movement of the examiner’s arm, as it could be the case in the kinetic boundary perimetry. Here, we emphasize the need of putting a patient in the condition to perform reliably. Therefore, we decided to ameliorate some procedural aspects to integrate and expand well-known common procedures, as described in the following paragraphs, where we sum up the changes made, provide a short rationale for their introduction, and briefly recap their helpfulness. The Scoresheet we provide (cf. Supplementary Materials), designed to facilitate the recording of patients’ responses, nicely complements the present study.

### 4.1. Changes to Existing Procedures

#### 4.1.1. Kinetic Boundary Perimetry Manoeuvring the Rod from A Rear Position with the Help of An Assistant Examiner

The setting usually adopted (e.g., [7]) when using a kinetic red target was modified. We carried out this gross perimetry by standing behind (instead of in front of) the patient, cooperating with an assistant examiner who carefully checked that the patient maintained their gaze on the fixation point. Testing patients from a rear position enhances response reliability, by preventing the possibility that they could naively respond based on an unintentional perception of the starting position of the rod on which the red target is mounted. One may argue that the involvement of an assistant examiner represents an unnecessary complication in the testing procedure. Conversely, we argue that such a change represents a reasonable trade-off between a slighter complication (which can be easily fixed with some good planning) and a substantially increased performance reliability. This is particularly true with stroke patients, who—as we have seen above—may be particularly inclined to be non-compliant with instructions due to their concern about their health condition, fear of failing tests, desire to “please” the examiner, etc. Indeed, we suggest that such an additional layer of control is what made the results of the Kinetic boundary perimetry so robust in the present work.

#### 4.1.2. More Trials in the Static Finger Wiggle Tests

The number of trials was expanded with respect to recommended or standard procedures that are normally based on very few trials (e.g., four overall trials per eye as in [41] for the finger counting test; two left, two right, and two bilateral trials, totalling six binocular trials overall, as in [30,31], to detect hemianopia or extinction with *static finger wiggle test* by hemifields in stroke patients). In the present study, each quadrant or hemifield assessed by *finger wiggle tests* was stimulated by default using 3 left, 3 right, and 3 bilateral trials in the *monocular finger wiggle test* by quadrants (totalling 36 trials); and 5 left, 5 right, and 5 bilateral trials in the binocular test by hemifields (totalling 15 trials). In addition, catch trials were used (see Section 4.1.3). Indeed, trials were repeated not only in the event of a loss of central fixation, but also in the event of unreliable responses (e.g., wiggling was reported for a catch trial). This latter case made it necessary to stimulate the visual field more times and repeat catch trials. This approach is essential when assessing RBD stroke patients because their attentional/motor deficits may interfere with the task. In addition, trials were added in further positions of the visual field, whenever necessary to refine the extension of a detected loss (e.g., [19]). Again, there is a trade-off between slightly longer testing times and a substantially increased performance reliability, and we believe it is worth the hassle. We look at it as a small investment with a substantial profit in return. All in all, the testing duration is still very reasonable (i.e., 3–7 min for the monocular test by quadrants).

#### 4.1.3. Catch Trials

As mentioned earlier, qualitative visual field tests may be challenging with RBD stroke patients, because of the possible presence of attentional/motor deficits beyond perceptual ones. The inclusion of catch trials randomly interspersed among regular trials serves two aims: (i) monitoring patients’ compliance, and hence improving performance reliability; and (ii) reassuring patients, so that they do not get anxious in trials in which they do not detect any target (further improving performance reliability), as they know that sometimes no target is presented. As discussed in the previous section, there is again a trade-off between slightly longer testing times and an increased reliability. And once more, we believe it is worth including just a few additional trials in return for more reliable results.

#### 4.1.4. Testing Quadrants Rather Than Whole Hemifields

A *binocular finger wiggle test* stimulating the whole hemifields (instead of quadrants) was included in the set of qualitative tests. This test does not appear to be appropriate for stroke patients, since placing the targets approximately across the edge between the upper and lower quadrants could accidentally miss quadrantic deficits. For instance, in a patient with an inferior quadrantanopia, a wiggling occurring even slightly above the horizontal meridian of the visual field would miss the presence of this VHFD, and would return extinction (if present) in the spared upper quadrant. Therefore, both VHFDs and visual extinction should be assessed separately in the upper and lower quadrants. However, this test is typically adopted in many settings to assess both hemianopias and visual extinction. As an example, it has been used in multicentric studies on stroke patients designed to assess sensitivity and validate a comprehensive battery for spatial neglect [30,31], and widely used “as is” to detect extinction in several studies involving stroke patients with neglect, without any formal assessment of visual field deficits (e.g., [42,43]). Therefore, despite we do not recommend adopting this procedure, we thought it was important to test its accuracy in the present study.

#### 4.1.5. Using Eye Patches

Most stroke patients cannot effectively cover their eyes with their hands due to the common presence of hemiplegia or hemiparesis. Therefore, in the present study, using an eye patch to occlude the non-tested eye was a necessary consequence of patients’ conditions. In our opinion, however, eye patches should be used even with patients who do not present with motor deficits. Eye patches not only prevent involuntary peeking or mistakenly covering the “wrong” eye, but also ensure homogeneous testing conditions across sessions and patients, and prevent potential pressing of the eyeball, which may hamper further examination of an eye. Unfortunately, asking patients to use their hand to cover the non-tested eye seems to be common practice in several studies, web video tutorials, and guidelines (e.g., [41,44,45]). We acknowledge that this choice may be due to the fact that patients coming from ophthalmology units are unlikely to present with hemiplegia/hemiparesis. In some other cases, a formal description of the procedure adopted is lacking and is replaced with a graphical illustration, e.g., showing a patient using their hand to cover an eye. Our hope is that such illustrations are simply a schematic, simplified portrayal that do not necessarily correspond exactly to what was actually done by practitioners. However, if these are true, then such examples would be highly misleading, and would not allow replicating the method at hand. Anyway, in our opinion, covering a patient’s eye with their hand should be a discouraged practice and using eye patches should be encouraged instead, to ensure more reliable responses and more homogeneous testing conditions.

### 4.2. Limitations

The present study assessed only RBD stroke patients, a limitation to be acknowledged. Left-brain-damaged (LBD) patients could not be included because this study is part of a larger study specifically recruiting and investigating RBD patients. However, homonymous hemianopias and quadrantanopias are significantly less frequent after left than right brain stroke (e.g., [3,4]). In addition, LBD patients could potentially present with aphasia, which implies the risk of hindering the comprehension of instructions and adherence to task demands, as well as hampering the verbal output. We can presume that, with minimal adjustments, the procedures outlined in this study can be successfully adopted with LBD patients as well, i.e., with right hemifield losses. Indeed, the literature on SAP [46] and confrontation tests (e.g., [17] does not report or recommend differential procedures for the assessment of right vs. left visual field deficits, nor does it suggest any different interpretation of their results. Only the potential presence of neglect in RBD patients is typically considered, i.e., the reason why double simultaneous stimulation is part of the finger wiggling tests (e.g., [31]). However, future studies should face the obstacle of potential concurrent language disorders, assess the accuracy of qualitative visual field tests in LBD patients, and determine whether the present procedures are suitable for these patients, or whether they need any adjustment.

Another limitation is that our study has little data to characterise the performance and reliability of qualitative tests in hemianopic stroke patients without neglect. However, this is not due to a weakness in our design, but rather to the fact that we were enrolling consecutive patients, who show the same characteristics as the target population, whereby the fraction of hemianopic patients without neglect is very small. This is probably due to the fact that strokes in the middle cerebral artery (which may determine both neglect and visual deficits) are more frequent than strokes in the posterior cerebral artery [47] (which normally affect only vision). Based on logical considerations supported by the literature, we would expect that qualitative tests should not be less accurate in patients without neglect with respect to patients with neglect. In fact, the sensitivity of qualitative tests relies on large and dense visual field losses (e.g., [17]), like the ones occurring in stroke patients (with or without neglect), and not in patients with pre-chiasmatic lesions. Therefore, indeed, we would expect quite the opposite, i.e., that qualitative tests perform better in non-neglect hemianopic patients, given the absence of attentional impairments. However, we acknowledge that further empirical evidence is needed to support the above-mentioned considerations.

## 5. Conclusions

The aim of the present study was to determine the sensitivity and specificity of four qualitative visual field tests in stroke patients. We also introduced a number of procedural ameliorations to enhance the reliability of performance in RBD stroke patients (who potentially present with attentional and/or motor deficits), but that we believe can be profitably adopted with other categories of patients as well. To the best of our knowledge, this is the first study addressing sensitivity and specificity of qualitative visual field tests entirely in a sample of stroke patients, therefore bridging a gap in the literature. Our results indicate that using a combination of the *monocular static finger wiggle test* and *kinetic boundary perimetry* yields the highest sensitivity, specificity, PPV and NPV, while keeping testing times within a reasonable range. This means that when either of these two tests detects a deficit, the patient is truly affected by a VHFD (100% PPV), while when neither test detects a deficit, the patient is truly not affected (100% NPV). The Scoresheet we devised to record patients’ responses (see Supplementary Materials) nicely complements the present study. We hope it will facilitate other researchers and practitioners’ data recording, and help standardising the administration and coding of results.

## Figures and Tables

**Table 1:** Demographic and clinical data of the whole sample of right-brain-damaged (RBD) stroke patients. VHFDs (visual hemifield deficits) refer to the standard automated perimetry (SAP; reference standard) outcome.

n	67
Sex	38 M; 29 F
Age (years)	61.5 (14.4)
Education (years)	11.7 (4.7)
Time since stroke (days)	57.4 (48.6)
N ischaemic stroke	46
N haemorrhagic stroke	21
**Patients with a VHFD**	**23**
Left homonymous hemianopia	18
Left homonymous inferior quadrantanopia	4
Left homonymous superior quadrantanopia	1
With neglect	21
Without neglect	2
**Patients without aVHFD**	**44**
With neglect	23
Without neglect	21

## Data Availability

The data presented in this study are available on request from the corresponding author. The data are not publicly available due to privacy policies.

## Supplementary Materials

The following supporting information can be downloaded at: https://www.mdpi.com/article/10.3390/brainsci14030235/s1, Supplementary Methods, Results, and Discussion, Supplementary Table S1, Supplementary Table S2, and Supplementary Table S3, De Luca et al. Supplementary Materials [46,47,48,49,50,51,52,53,54,55,56,57,58,59]; Scoresheet: De Luca et al. Scoresheet for qualitative visual field tests.
