# Supplementary material for: Sensitivity and Specificity of Qualitative Visual Field Tests for Screening Visual Hemifield Deficits in Right-Brain-Damaged Stroke Patients
Source: Brain Sci. 2024 Feb 29;14(3):235. doi: 10.3390/brainsci14030235 (PMC10969102; doi:10.3390/brainsci14030235)
Supplement: Supplementary file 1 [file brainsci-14-00235-s001.zip › De Luca et al Brain Sci 2024 Scoresheet for qualitative visual field tests.pdf]

QUALITATIVE VISUAL FIELD TESTS SCORESHEET

Supplementary Material to the paper "Sensitivity and specificity of qualitative visual field tests for screening visual hemifield deficits in right-brain-damaged stroke patients" by De Luca et al.

The order of the four tests can be randomized based on the aim of the assessment, e.g. whether for research purposes, or clinical screening for visual hemifield deficits.
For each test, the examiner indicates the outcome as either a '*present*' or '*absent*' deficit.
Abbreviations: RE = right eye; LE = left eye.

Date: ___________________________ Patient ID: ____

Examiner ID: _____________________ Notes: ____

____

____

Face description test

1 The examiner sits on an adjustable seat in front of the patient, ensuring that their eyes are at the same height. The eye-to-eye distance is approx. 65 cm (see Figure 1a in the paper). The examiner wears a red dot patch (0.5 cm diameter) on the tip of the nose, which serves as a fixation point.
2 The right and the left eyes are examined separately, with the non-tested eye occluded using an eye patch.
3 The examiner asks the patient to indicate whether they could see their face clearly, or whether any part of it appears blurred or missing.

Outcome:

RE	LE

Notes: ____

____

Binocular static finger wiggle test

1. The examiner sits on an adjustable seat in front of the patient, ensuring that their eyes are at the same height. The eye-to-eye distance is approx. 65 cm. The examiner wears a red dot patch (0.5 cm diameter) on the tip of the nose, which serves as a fixation point.
2. The examiner widens their arms and places the fists (with the indexes pointing upwards) in the frontal plane, halfway between themselves and the patient, at the height of the horizontal meridian of the visual field (see Figure 1b in the paper). The examiner makes sure to see the tip of their indexes while looking at the patient (each target is approx. 50 deg of eccentricity relative to fixation). Both index fingers are always present on either side with respect to the vertical meridian.
3. The finger could wiggle either on one side (right or left), bilaterally (to assess the possible presence of visual extinction), or not at all (catch trials, for performance reliability).
4. To start the test, the examiner asks the patient to keep their gaze steady on the fixation point, and report on which side the finger(s) moved (right, left, both, or none). The examiner explains that sometimes no finger will wiggle when asking for a response.
5. The patient is instructed to respond based on their perspective, using a few training trials before the actual examination begins.
6. The first target is presented to the right visual hemifield of the patient, in order to start off with a stimulus likely to be detected. Then, all four conditions are randomly tested at least five times each.
7. Any response provided following a loss of central fixation is discarded, and the trial is repeated in random order later on.
8. For each trial, the examiner assigns a score to the patient's response by filling the SCORING TABLE below in, according to the SCORING LEGEND on the right. '**+**' indicates a correct response, '**-**' indicates a missing target(s), and '**L**' and '**R**' indicate when the patient responds 'left' and 'right' erroneously, respectively.
9. The Outcome box under the SCORING TABLE is filled in with either '*present*' or '*absent*'. In the case of absence of visual hemifield deficits, the examiner indicates the presence or absence of extinction.

SCORING TABLE

**Trial condition**	**Scores**
UNILATERAL (left)	
UNILATERAL (right)	
BILATERAL	
Catch trials	

SCORING LEGEND

**Trial condition**	**Patient's response**			
	Left	Right	Both	None
UNILAT. (left)	+	R	LR	-
UNILAT. (right)	L	+	LR	-
BILATERAL	L	R	+	-
Catch trials	L	R	LR	+

Outcome:

Notes:

____

Brain Sci. **2024**, *14*, 235. https://doi.org/10.3390/brainsci14030235 De Luca et al. Sensitivity and specificity of qualitative visual field tests for screening visual hemifield deficits in right-brain-damaged stroke patients

Monocular static finger wiggle test

1. The examiner sits on an adjustable seat in front of the patient, ensuring that their are at the same height. The eye-to-eye distance is approx. 65 cm. The examiner wears a red dot patch (0.5 cm diameter) on the tip of the nose, which serves as a fixation point.
2. The right and the left eyes are examined separately, with the non-tested eye occluded using an eye patch. When the patient's right eye is examined, the examiner closes their own left eye, and vice-versa.
3. The examiner widens their arms and places the fists (with the indexes pointing upwards) in the frontal plane, halfway between themselves and the patient, either in the upper or lower quadrants of the visual field (which are tested separately; see Figure 1c in the paper). The examiner makes sure to see the tip of their indexes while looking at the patient (each target is approx. 30 deg of eccentricity relative to fixation). Both index fingers are always present on either side with respect to the vertical meridian.
4. The finger could wiggle either on one side (right or left), bilaterally (to assess the possible presence of visual extinction), or not at all (catch trials, for performance reliability).
5. To start the test, the examiner asks the patient to keep their gaze steady on the fixation point, and report on which side the finger(s) moved (right, left, both, or none). The examiner explains that sometimes no finger will wiggle when asking for a response.
6. The patient is instructed to respond based on their perspective, using a few training trials before the actual examination begins.
7. The first target is presented to the right visual hemifield of the patient, in order to start off with a stimulus likely to be detected. Then, all four conditions are randomly tested at least three times each.
8. Any response provided following a loss of central fixation is discarded, and the trial is repeated in random order later on.
9. In the SCORING TABLES (cf. next page), the examiner assigns scores to each response, separately for the two eyes, and the upper and lower quadrants, in the designated cells for the four trial conditions. For each trial, the examiner fills the SCORING TABLES in, according to the SCORING LEGEND at the bottom of this page. '**+**' indicates a correct response, '**-**' indicates a missing target(s), and **'L'** and **'R'** indicate when the patient responds 'left' and 'right' erroneously, respectively.
10. The quadrants of the SCORING GRIDS (cf. next page) can be shaded, to summarize the results of the SCORING TABLES in case a visual hemifield deficit is present.
11. The Outcome box is filled in with either '*present*' or '*absent*'. In case a visual hemifield deficit is absent, the examiner indicates the presence or absence of extinction.

SCORING LEGEND

**Trial condition**	**Patient's response** Left	Right	Both	None
UNILAT. (left)	+	R	LR	-
UNILAT. (right)	L	+	LR	-
BILATERAL	L	R	+	-
Catch trials	L	R	LR	+

Monocular static finger wiggle test

Date: ____________________ Patient ID: ____

SCORING TABLES

RE upper quadrants

UNILATERAL (left)
UNILATERAL (right)
BILATERAL
Catch trials

LE upper quadrants

UNILATERAL (left)
UNILATERAL (right)
BILATERAL
Catch trials

RE lower quadrants

UNILATERAL (left)
UNILATERAL (right)
BILATERAL
Catch trials

LE lower quadrants

UNILATERAL (left)
UNILATERAL (right)
BILATERAL
Catch trials

SCORING GRIDS

RE

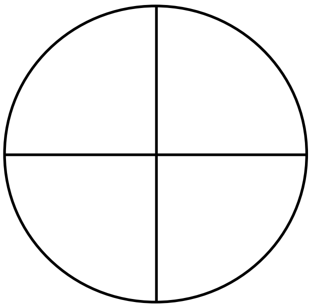

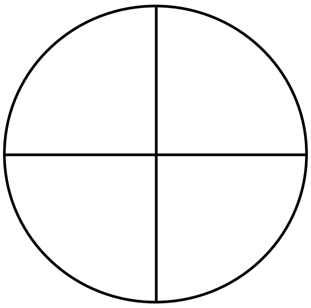

LE

Outcome:

RE	LE

Notes: ____

Brain Sci. **2024**, *14*, 235. https://doi.org/10.3390/brainsci14030235 De Luca et al. Sensitivity and specificity of qualitative visual field tests for screening visual hemifield deficits in right-brain-damaged stroke patients

Kinetic boundary perimetry (with a red target)

1. A chief examiner and an assistant examiner cooperate to conduct the test (see Figure 1d in the paper). The assistant examiner sits on an adjustable seat in front of the patient, ensuring that their eyes are at the same height. The assistant examiner wears a red dot patch (0.5 cm diameter) on the tip of the nose, which serves as a fixation point. The assistant examiner monitors the patient's maintenance of fixation and warns the chief examiner in case of invalid trials due to any displacement of the patient's eyes.
2. The right and the left eyes are examined separately, with the non-tested eye occluded using an eye patch.
3. The chief examiner stands behind the patient holding and manoeuvring a 33 cm long transparent stick with a 15 mm red sphere on one end which serves as the target (e.g., Aston Perimetry Tool). The chief examiner moves the tool so that the red target follows an arc trajectory from the periphery (90°, blue numbers in the grid in the figure below) to the centre of the patient's visual field along 8 meridians (0°, 45°, 90°, 135°, 180°, 225°, 270° and 315°; green numbers in the grid in the figure below). The arc is centred on the patient's eye at a radial distance of about 33 cm. The speed of the target is approx. 5-7 deg/sec.
4. The patient is required to maintain fixation by focussing on the fixation point, and report as soon as the target appears in their visual field (unseen-to-seen procedure). If necessary, the assistant examiner reminds the patient not to move their eyes, or to respond promptly when the target is detected.
5. Each trajectory is examined three times (or more in case of unreliable trials, or loss of central fixation). The 8 meridians are examined in a randomized sequence. Trials with unreliable responses or displacement of fixation should not be repeated consecutively.
6. The chief examiner records on the appropriate RE or LE SCORING GRIDS (cf. last page) the point where the patient detects the target. This is done by making a mark, approximately in the position where the patient reports the sudden detection. For further details, see Figure 1b in the paper.
7. For convenience, the examiner can use progressive numbers (1, 2, 3, etc.) to keep track of successive testing rounds for each meridian. Numbers (1,2,3) in the figure below represent examples of successive testing rounds of meridians 45° and 315°.
8. Additionally, the "from-seen-to-unseen" procedure can be applied for a more accurate assessment. The patient informs the examiner when the target disappears, and the examiner marks the corresponding positions with consecutive negative numbers (-1, -2, -3, etc.).

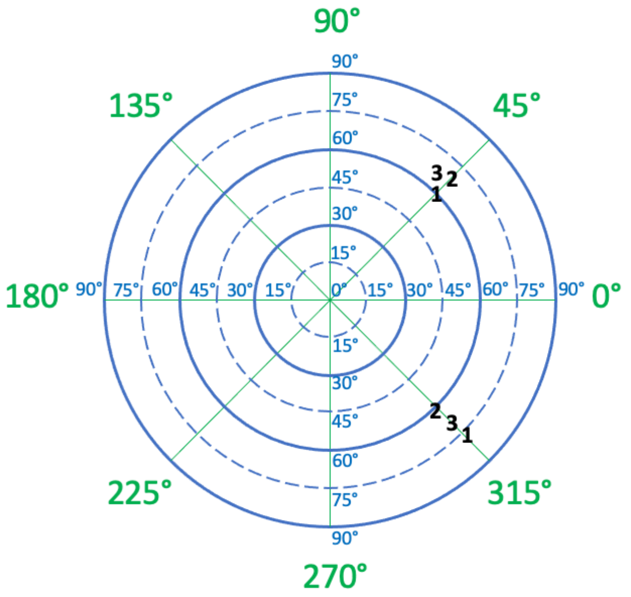

Kinetic boundary perimetry (with a red target)

Date: ______________________ Patient ID: _______________________________

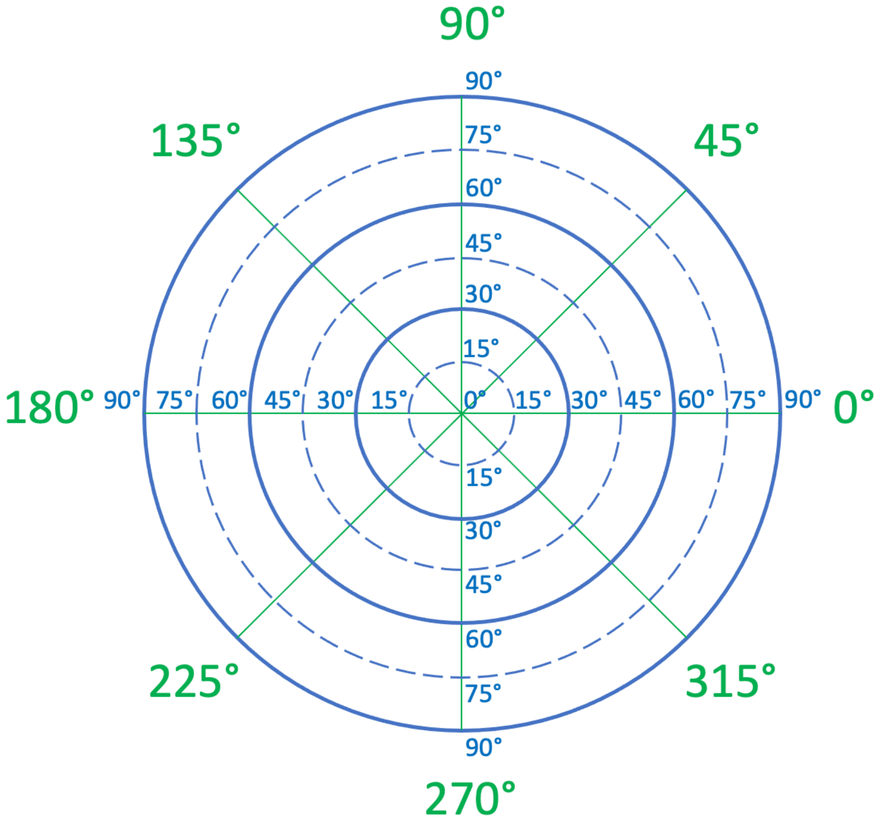

RE

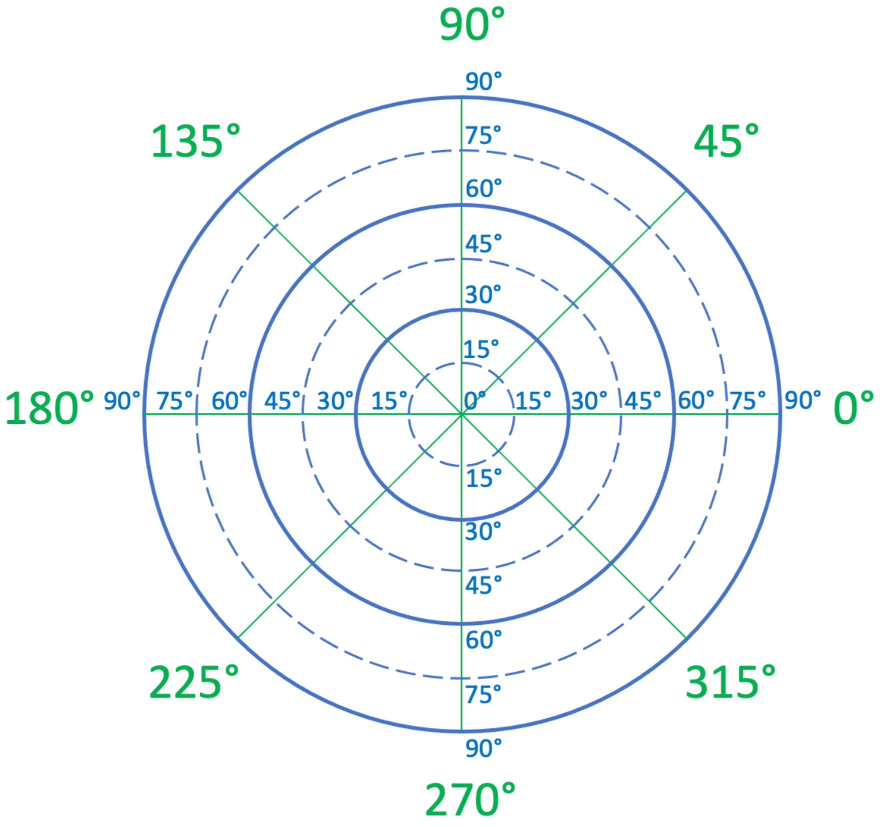

LE

Outcome:

RE	LE

Brain Sci. **2024**, *14*, 235. https://doi.org/10.3390/brainsci14030235 De Luca et al. Sensitivity and specificity of qualitative visual field tests for screening visual hemifield deficits in right-brain-damaged stroke patients
