# Supplementary material for: Sensitivity and Specificity of Qualitative Visual Field Tests for Screening Visual Hemifield Deficits in Right-Brain-Damaged Stroke Patients
Source: Brain Sci. 2024 Feb 29;14(3):235. doi: 10.3390/brainsci14030235 (PMC10969102; doi:10.3390/brainsci14030235)
Supplement: Supplementary file 1 [file brainsci-14-00235-s001.zip › De Luca et al Brain Sci 2024 Supplementary Materials.pdf]

Sensitivity and Specificity of Qualitative Visual Field Tests for Screening Visual Hemifield Deficits in Right-Brain-Damaged Stroke Patients

by Maria De Luca, Fabrizio Zeri, Alessandro Matano, Concetta Di Lorenzo, Maria Paola Ciurli, Martina Mulas, Virginia Pollarini, Stefano Paolucci, and Davide Nardo

Supplementary Methods

Neglect assessment

As part of a thorough neuropsychological assessment, the 67 right-brain damaged (RBD) patients underwent a battery for neglect that included cancellation [46–52], line bisection [53,54], perceptual [55], and sentence reading [50] tests, as well as tests for personal and extra-personal neglect [56] (see [32], for tests details and cut-offs). A spatial bias score was computed for cancellation tests, representing the deficit in terms of the number of left- minus right-side target omissions regardless of targets' spatial distribution (e.g., [31,54]). Patients failing at least two tests (e.g., cf. [30,31,57]) were classified as having neglect (N+). If they did not fail any test or failed only one test, patients were classified as not having neglect (N–). As a result of the neglect battery, there were 44 N+ and 23 N– patients (see Table 1 in the paper).

Neglect severity (classified as: borderline, mild, moderate, or severe; see the corresponding column in Supplementary Table S1) was determined by the senior neuropsychologists who administered the tests and interpreted the neglect battery results (A.M. and M.P.C.). There were 8 patients with borderline, 10 with mild, 12 with moderate, and 14 with severe neglect (see Supplementary Table S2). Additionally, for Letter [48,50], Star [51,52], and Line cancellation [46,50] tests, the *Center of Cancellation score* (CoC; [58]) was also computed using the purpose-built software (https://github.com/neurolabusc/Cancel). Differently from the spatial bias score, the CoC measures the cancellation performance as a continuous measure, constituting a more accurate measure of neglect severity. The CoC score ranges from –1.00 to +1.00, whereby 0.00 indicates symmetrical performance, and +1.00 indicates detection of the rightmost item only. Importantly, patients' neglect severity as determined by the neuropsychologists was consistent with the continuous severity score measured by the CoC (averages reported in Supplementary Table S2, separately for each level of severity).

Supplementary Results

Possible association between neglect severity and the presence of a VHFD

To examine the possible association between neglect severity and the presence of a VHFD, a χ^2 test was carried out based on the number of neglect patients with or without a VHFD and neglect severity values (cf. "N+ with VHFD" and "N+ without VHFD" columns in Supplementary Table S2). To do so, we merged borderline and mild N+ patients on the one hand, and moderate and severe N+ patients on the other. The test showed that there was no significant association between neglect severity and presence of a VHFD ($\chi^2 = 0.132$, $p = .717$).

Sensitivity and specificity of the qualitative visual field assessment in N+ patients

The sensitivity, specificity, PPV, NPV and 95% CI was determined separately for this subsample of 44 neglect patients, since they contributed with most VHFD to the whole sample (see Table 1 in the paper, and Supplementary Table S1). Results are presented in Supplementary Table S3 (cf. Table S3a for individual tests, and Table S3b for their combinations). Importantly, results in the subsample replicated those obtained in the whole sample, The best individual tests were the *kinetic boundary perimetry* and

Brain Sci. **2024**, *14*, 235. https://doi.org/10.3390/brainsci14030235 De Luca et al. Sensitivity and specificity of qualitative visual field tests for screening visual hemifield deficits in right-brain-damaged stroke patients

monocular finger wiggle test (sensitivity = 90.5%). When the *kinetic boundary perimetry* was combined with any of the other tests, the number of true positive cases improved, reaching a sensitivity of 100% for some combinations (see Figure 2 in the paper).

Supplementary Discussion

Given that almost all patients who exhibited a VHFD in the present study were neglect patients, we recomputed the accuracy of qualitative visual field tests net of N– patients, who mostly contributed with negative cases. Of note, the pattern of results of the four metrics of accuracy (Supplementary Table S3) nicely overlapped with the results obtained in the whole sample (Table 2 in the paper). This was expected, as N– patients only contributed with 2 VHFD out of 23 N– patients, while N+ patients with 21 VHFD out of 23.

It has been established that homonymous hemianopias are more frequent in stroke patients with a right-brain damage (RBD) than in those with a left-brain damage (LBD) (e.g., [3,4]). One may wonder whether these findings are due to the presence of neglect patients in stroke samples, with their neglect symptoms masquerading as a VHFD (e.g., [59]). It is not in the scope of the present work to disentangle the debate about whether neglect negatively affects primary sensory deficits (e.g., [59]), or vice-versa (e.g., [23]). The above-mentioned studies [3,4] examined more than a thousand patients, and separately classified them according to the presence/absence of neglect. It is well known that neglect and visual field deficits may co-occur, or exist independently from each other. Indeed, a double dissociation has been demonstrated between sensory and attentional impairment, but the two deficits more often co-occur [23]. Presumably, the close association between neglect and VHFD, which are functionally distinct, is due to the anatomical contiguity of sites damaged by the stroke [25]. Hence, neglect and a primary sensory deficit are more likely to co-occur when a retro-chiasmatic cortical lesion causing neglect extends into the occipital lobe and/or the underlying white matter. Indeed, it has been reported that the prevalence of visual field deficits in neglect patients could be more than 50% (e.g., [25]).

Anyway, it is worth noting that the present study included several patients with moderate and severe neglect who did not show any VHFD, both at the standard automated perimetry (SAP), and at qualitative visual field tests. In fact, about 2/3 of patients with moderate neglect did not exhibit any primary sensory deficit. Borderline neglect patients showed a similar figure (i.e., 2/3), while mild and severe neglect patients showed about 1/3 and 1/2, respectively. The results of the present study are consistent with the common agreement that neglect patients diagnosed with a VHFD are affected by an actual visual impairment, despite the potential confounding role played by neglect. Indeed, despite the attentional problems of these patients, a standard automated perimetry can be reliably performed and typically returns clear grids of dense homogeneous visual field losses, with a clear-cut vertical meridian (in case of hemianopia), or both a horizontal and a vertical meridian (in case of quadrantanopia).

Supplementary Table S1. Demographic and clinical information and results of the qualitative visual field tests by participants. Demographic and clinical data are reported alongside the classification of patients into true/false positive/negative cases. Left visual hemifield deficits (VHFD) diagnosed by the standard automated perimetry (SAP) are homonymous hemianopia (labelled as "hemianopia"), homonymous inferior quadrantanopia ("inferior Q"), or homonymous superior quadrantanopia ("superior Q"). N+ and N– refer to patients with or without neglect, respectively. True positive and negative cases are labelled as "TRUE +" and "true –", respectively. False negative cases are labelled as "false –". There were no false positive cases. Separately for the four individual tests (Face description, abbreviated as "F"; Binocular static finger wiggle test, "B"; Monocular static finger wiggle test, "M"; Kinetic boundary perimetry, "K") and their eleven combinations, the classification into true/false positive/negative is determined by comparing the qualitative visual field test binary outcome (i.e., the presence or absence of a VHFD) with the actual VHFD as diagnosed by the SAP. For the monocular static finger wiggle test and the kinetic boundary perimetry, the characterisation of the type of deficit was also presented, specifying whether the VHFD detected by the qualitative test was the same ("yes"), was different (mismatch), or returned an incongruous result with respect to the type of VHFD diagnosed by the SAP. In case of a mismatch, the type of deficit detected by the qualitative test is indicated in parentheses. Incongruos results between the eyes were specified (LE = left eye; RE = right eye). For extinction, the symbol "–" indicates that the symptom was neither present nor absent, since left stimuli were not perceived when presented alone (i.e., a VHFD was present); "yes/–" indicates that extinction was present in the quadrant that was always perceived during unilateral stimulation, while the other quadrant was not perceived under unilateral stimulation (i.e., a VHFD was detected in that quadrant); "no/–" indicates that extinction was not present in the quadrant that was always perceived during unilateral stimulation, while the other quadrant was not perceived under unilateral stimulation (i.e., a VHFD was detected).

Demographic and clinical information									Individual qualitative visual field tests compared with SAP				Combinations of qualitative visual field tests compared with SAP											Characterisation of the type of deficit		Extinction	
									1 test				2-test						3-test				4-test				
Patient ID	Sex	Age (yrs)	Education (yrs)	Aetiology	Time since stroke (days)	Left VHFD based on SAP outcome	Neglect outcome	Neglect severity	F	B	M	K	F + B	F + M	F + K	B + M	B + K	M + K	F + B + M	F + B + K	F + M + K	B + M + K	F + B + M + K	M detected the same deficit as SAP?	K detected the same deficit as SAP?	B	M
1	M	75,5	5	ischaemia	25	hemianopia	N+	borderline	false –	false –	TRUE +	TRUE +	false –	TRUE +	TRUE +	TRUE +	TRUE +	TRUE +	TRUE +	TRUE +	TRUE +	TRUE +	TRUE +	mismatch (superior Q)	incongruous (RE hemianop., LE inferior Q)	no	no/–
2	F	36,6	18	haemorrhage	69	hemianopia	N+	borderline	false –	false –	false –	TRUE +	false –	false –	TRUE +	false –	TRUE +	TRUE +	false –	TRUE +	TRUE +	TRUE +	TRUE +	false negative	mismatch (inferior Q)	no	yes (in sup. quadrant)
3	M	38,0	13	haemorrhage	42	hemianopia	N+	borderline	false –	TRUE +	TRUE +	TRUE +	TRUE +	TRUE +	TRUE +	TRUE +	TRUE +	TRUE +	TRUE +	TRUE +	TRUE +	TRUE +	TRUE +	yes	yes	–	–
4	M	76,9	18	ischaemia	86	hemianopia	N+	mild	false –	TRUE +	TRUE +	TRUE +	TRUE +	TRUE +	TRUE +	TRUE +	TRUE +	TRUE +	TRUE +	TRUE +	TRUE +	TRUE +	TRUE +	yes	yes	–	–
8	M	55,3	8	ischaemia	26	hemianopia	N+	mild	false –	TRUE +	TRUE +	TRUE +	TRUE +	TRUE +	TRUE +	TRUE +	TRUE +	TRUE +	TRUE +	TRUE +	TRUE +	TRUE +	TRUE +	incongruous (RE hemianop., LE superior Q)	incongr. (RE hemianop., LE superior Q)	–	yes (in inf. quadrant) / –
6	F	79,5	5	ischaemia	35	hemianopia	N+	moderate	false –	TRUE +	TRUE +	TRUE +	TRUE +	TRUE +	TRUE +	TRUE +	TRUE +	TRUE +	TRUE +	TRUE +	TRUE +	TRUE +	TRUE +	yes	yes	–	–
10	M	61,5	13	ischaemia	161	hemianopia	N+	moderate	false –	TRUE +	TRUE +	false –	TRUE +	TRUE +	false –	TRUE +	TRUE +	TRUE +	TRUE +	TRUE +	TRUE +	TRUE +	TRUE +	yes	false negative	–	–
13	M	66,6	13	ischaemia	37	hemianopia	N+	moderate	false –	false –	TRUE +	TRUE +	false –	TRUE +	TRUE +	TRUE +	TRUE +	TRUE +	TRUE +	TRUE +	TRUE +	TRUE +	TRUE +	mismatch (inferior Q)	yes	no	yes (in sup. quadrant) / –
5	M	32,5	8	haemorrhage	44	hemianopia	N+	severe	false –	TRUE +	TRUE +	TRUE +	TRUE +	TRUE +	TRUE +	TRUE +	TRUE +	TRUE +	TRUE +	TRUE +	TRUE +	TRUE +	TRUE +	yes	yes	–	–
7	F	73,5	12	ischaemia	39	hemianopia	N+	severe	false –	false –	false –	TRUE +	false –	false –	TRUE +	false –	TRUE +	TRUE +	false –	TRUE +	TRUE +	TRUE +	TRUE +	false negative	yes	yes	yes
9	F	65,8	8	haemorrhage	115	hemianopia	N+	severe	TRUE +	TRUE +	TRUE +	false –	TRUE +	TRUE +	TRUE +	TRUE +	TRUE +	TRUE +	TRUE +	TRUE +	TRUE +	TRUE +	TRUE +	yes	false negative	–	–
11	M	53,0	13	ischaemia	226	hemianopia	N+	severe	false –	TRUE +	TRUE +	TRUE +	TRUE +	TRUE +	TRUE +	TRUE +	TRUE +	TRUE +	TRUE +	TRUE +	TRUE +	TRUE +	TRUE +	yes	yes	–	–
12	F	41,7	18	haemorrhage	76	hemianopia	N+	severe	false –	TRUE +	TRUE +	TRUE +	TRUE +	TRUE +	TRUE +	TRUE +	TRUE +	TRUE +	TRUE +	TRUE +	TRUE +	TRUE +	TRUE +	yes	yes	–	–
14	M	66,3	13	ischaemia	22	hemianopia	N+	severe	false –	TRUE +	TRUE +	TRUE +	TRUE +	TRUE +	TRUE +	TRUE +	TRUE +	TRUE +	TRUE +	TRUE +	TRUE +	TRUE +	TRUE +	yes	yes	–	–
15	M	55,7	5	ischaemia	43	hemianopia	N+	severe	false –	TRUE +	TRUE +	TRUE +	TRUE +	TRUE +	TRUE +	TRUE +	TRUE +	TRUE +	TRUE +	TRUE +	TRUE +	TRUE +	TRUE +	yes	mismatch (inferior Q)	no	no
16	M	67,7	8	ischaemia	21	hemianopia	N+	severe	TRUE +	TRUE +	TRUE +	TRUE +	TRUE +	TRUE +	TRUE +	TRUE +	TRUE +	TRUE +	TRUE +	TRUE +	TRUE +	TRUE +	TRUE +	yes	yes	no	no
17	M	51,8	18	haemorrhage	71	inferior Q	N+	mild	false –	false –	TRUE +	TRUE +	false –	TRUE +	TRUE +	TRUE +	TRUE +	TRUE +	TRUE +	TRUE +	TRUE +	TRUE +	TRUE +	yes	yes	yes	yes (in sup. quadrant) / –
18	F	41,4	13	ischaemia	52	inferior Q	N+	mild	false –	false –	TRUE +	TRUE +	false –	TRUE +	TRUE +	TRUE +	TRUE +	TRUE +	TRUE +	TRUE +	TRUE +	TRUE +	TRUE +	yes	yes	yes	yes (in sup. quadrant) / –
19	M	58,1	18	ischaemia	40	inferior Q	N+	mild	false –	false –	TRUE +	TRUE +	false –	TRUE +	TRUE +	TRUE +	TRUE +	TRUE +	TRUE +	TRUE +	TRUE +	TRUE +	TRUE +	yes	yes	yes	yes (in sup. quadrant) / –
20	F	48,3	13	haemorrhage	70	inferior Q	N+	severe	false –	false –	TRUE +	TRUE +	false –	TRUE +	TRUE +	TRUE +	TRUE +	TRUE +	TRUE +	TRUE +	TRUE +	TRUE +	TRUE +	yes	yes	yes	yes (in sup. quadrant) / –
21	F	43,8	16	ischaemia	43	superior Q	N+	moderate	false –	TRUE +	TRUE +	TRUE +	TRUE +	TRUE +	TRUE +	TRUE +	TRUE +	TRUE +	TRUE +	TRUE +	TRUE +	TRUE +	TRUE +	yes	yes	–	no (in inf. quadrant) / –
22	M	64,8	11	ischaemia	43	hemianopia	N–	-	false –	false –	false –	TRUE +	false –	false –	TRUE +	false –	TRUE +	TRUE +	false –	TRUE +	TRUE +	TRUE +	TRUE +	false negative	mismatch (inferior Q)	no	no
23	M	81,4	8	ischaemia	26	hemianopia	N–	-	TRUE +	TRUE +	TRUE +	TRUE +	TRUE +	TRUE +	TRUE +	TRUE +	TRUE +	TRUE +	TRUE +	TRUE +	TRUE +	TRUE +	TRUE +	yes	yes	–	–
24	M	57,2	18	ischaemia	112	none	N+	borderline	true –	true –	true –	true –	true –	true –	true –	true –	true –	true –	true –	true –	true –	true –	true –			no	yes
25	M	47,8	13	ischaemia	303	none	N+	borderline	true –	true –	true –	true –	true –	true –	true –	true –	true –	true –	true –	true –	true –	true –	true –			no	no
26	M	85,2	8	haemorrhage	35	none	N+	borderline	true –	true –	true –	true –	true –	true –	true –	true –	true –	true –	true –	true –	true –	true –	true –			no	no
28	F	50,0	13	haemorrhage	42	none	N+	borderline	true –	true –	true –	true –	true –	true –	true –	true –	true –	true –	true –	true –	true –	true –	true –			no	no
41	F	78,3	5	ischaemia	28	none	N+	borderline	true –	true –	true –	true –	true –	true –	true –	true –	true –	true –	true –	true –	true –	true –	true –			no	yes
27	F	51,6	8	ischaemia	29	none	N+	mild	true –	true –	true –	true –	true –	true –	true –	true –	true –	true –	true –	true –	true –	true –	true –			no	yes
30	M	73,4	11	haemorrhage	77	none	N+	mild	true –	true –	true –	true –	true –	true –	true –	true –	true –	true –	true –	true –	true –	true –	true –			no	no
32	F	75,6	13	ischaemia	38	none	N+	mild	true –	true –	true –	true –	true –	true –	true –	true –	true –	true –	true –	true –	true –	true –	true –			no	yes
34	M	73,7	6	ischaemia	31	none	N+	mild	true –	true –	true –	true –	true –	true –	true –	true –	true –	true –	true –	true –	true –	true –	true –			no	no
36	F	46,5	8	ischaemia	91	none	N+	mild	true –	true –	true –	true –	true –	true –	true –	true –	true –	true –	true –	true –	true –	true –	true –			yes	yes
29	M	76,4	8	haemorrhage	110	none	N+	moderate	true –	true –	true –	true –	true –	true –	true –	true –	true –	true –	true –	true –	true –	true –	true –			yes	yes
31	F	67,4	17	haemorrhage	85	none	N+	moderate	true –	true –	true –	true –	true –	true –	true –	true –	true –	true –	true –	true –	true –	true –	true –			yes	yes
33	M	49,0	8	haemorrhage	61	none	N+	moderate	true –	true –	true –	true –	true –	true –	true –	true –	true –	true –	true –	true –	true –	true –	true –			no	no
35	M	48,6	18	ischaemia	51	none	N+	moderate	true –	true –	true –	true –	true –	true –	true –	true –	true –	true –	true –	true –	true –	true –	true –			no	no
37	F	80,7	5	ischaemia	34	none	N+	moderate	true –	true –	true –	true –	true –	true –	true –	true –	true –	true –	true –	true –	true –	true –	true –			no	no
40	F	61,5	5	ischaemia	33	none	N+	moderate	true –	true –	true –	true –	true –	true –	true –	true –	true –	true –	true –	true –	true –	true –	true –			no	no
43	M	60,7	8	ischaemia	27	none	N+	moderate	true –	true –	true –	true –	true –	true –	true –	true –	true –	true –	true –	true –	true –	true –	true –			yes	yes
44	M	77,4	18	haemorrhage	36	none	N+	moderate	true –	true –	true –	true –	true –	true –	true –	true –	true –	true –	true –	true –	true –	true –	true –			no	no
38	F	69,0	5	ischaemia	45	none	N+	severe	true –	true –	true –	true –	true –	true –	true –	true –	true –	true –	true –	true –	true –	true –	true –			yes	no
39	M	54,0	18	haemorrhage	27	none	N+	severe	true –	true –	true –	true –	true –	true –	true –	true –	true –	true –	true –	true –	true –	true –	true –			no	no
42	F	64,3	11	haemorrhage	17	none	N+	severe	true –	true –	true –	true –	true –	true –	true –	true –	true –	true –	true –	true –	true –	true –	true –			no	no
45	M	56,8	8	ischaemia	22	none	N+	severe	true –	true –	true –	true –	true –	true –	true –	true –	true –	true –	true –	true –	true –	true –	true –			yes	yes
46	F	64,1	18	ischaemia	126	none	N+	severe	true –	true –	true –	true –	true –	true –	true –	true –	true –	true –	true –	true –	true –	true –	true –			yes	yes
47	M	60,4	18	haemorrhage	83	none	N–	-	true –	true –	true –	true –	true –	true –	true –	true –	true –	true –	true –	true –	true –	true –	true –			no	no
48	F	55,9	8	ischaemia	134	none	N–	-	true –	true –	true –	true –	true –	true –	true –	true –	true –	true –	true –	true –	true –	true –	true –			no	no
49	M	61,2	18	haemorrhage	34	none	N–	-	true –	true –	true –	true –	true –	true –	true –	true –	true –	true –	true –	true –	true –	true –	true –			no	no
50	F	78,8	13	ischaemia	63	none	N–	-	true –	true –	true –	true –	true –	true –	true –	true –	true –	true –	true –	true –	true –	true –	true –			no	no
51	F	75,2	9	ischaemia	73	none	N–	-	true –	true –	true –	true –	true –	true –	true –	true –	true –	true –	true –	true –	true –	true –	true –			no	no
52	M	61,1	13	ischaemia	41	none	N–	-	true –	true –	true –	true –	true –	true –	true –	true –	true –	true –	true –	true –	true –	true –	true –			no	no
53	M	74,8	8	haemorrhage	36	none	N–	-	true –	true –	true –	true –	true –	true –	true –	true –	true –	true –	true –	true –	true –	true –	true –			no	no
54	F	73,1	8	ischaemia	31	none	N–	-	true –	true –	true –	true –	true –	true –	true –	true –	true –	true –	true –	true –	true –	true –	true –			no	no
55	M	71,0	18	ischaemia	38	none	N–	-	true –	true –	true –	true –	true –	true –	true –	true –	true –	true –	true –	true –	true –	true –	true –			no	no
56	F	74,9	5	ischaemia	72	none	N–	-	true –	true –	true –	true –	true –	true –	true –	true –	true –	true –	true –	true –	true –	true –	true –			no	no
57	M	69,6	5	ischaemia	25	none	N–	-	true –	true –	true –	true –	true –	true –	true –	true –	true –	true –	true –	true –	true –	true –	true –			no	no
58	F	87,5	13	ischaemia	19	none	N–	-	true –	true –	true –	true –	true –	true –	true –	true –	true –	true –	true –	true –	true –	true –	true –			no	no
59	F	70,9	5	ischaemia	24	none	N–	-	true –	true –	true –	true –	true –	true –	true –	true –	true –	true –	true –	true –	true –	true –	true –			no	no
60	F	67,3	13	ischaemia	30	none	N–	-	true –	true –	true –	true –	true –	true –	true –	true –	true –	true –	true –	true –	true –	true –	true –			no	no
61	M	60,0	16	ischaemia	31	none	N–	-	true –	true –	true –	true –	true –	true –	true –	true –	true –	true –	true –	true –	true –	true –	true –			no	no
62	M	65,0	18	ischaemia	36	none	N–	-	true –	true –	true –	true –	true –	true –	true –	true –	true –	true –	true –	true –	true –	true –	true –			no	no
63	M	30,9	18	ischaemia	19	none	N–	-	true –	true –	true –	true –	true –	true –	true –	true –	true –	true –	true –	true –	true –	true –	true –			no	no
64	M	72,3	18	ischaemia	29	none	N–	-	true –	true –	true –	true –	true –	true –	true –	true –	true –	true –	true –	true –	true –	true –	true –			no	no
65	F	45,4	8	haemorrhage	42	none	N–	-	true –	true –	true –	true –	true –	true –	true –	true –	true –	true –	true –	true –	true –	true –	true –			no	no
66	F	39,5	12	ischaemia	26	none	N–	-	true –	true –	true –	true –	true –	true –	true –	true –	true –	true –	true –	true –	true –	true –	true –			no	no
67	M	21,1	15	haemorrhage	72	none	N–	-	true –	true –	true –	true –	true –	true –	true –	true –	true –	true –	true –	true –	true –	true –	true –			no	no

Supplementary Table S2. Neglect severity is reported for the subsample of right-brain-damaged (RBD) stroke patients with neglect (n = 44). The number of neglect (N+) patients is reported in the second column separately for the four levels of neglect severity. The next columns report the number of N+ patients with or without a visual hemifield deficit (N+ with VHFD and N+ without VHFD, respectively). The last three columns report the results (i.e., mean ± SD) of a quantitative continuous measure of neglect severity, the *Center of Cancellation score* (CoC; [58]), ranging from –1.00 to +1.00, whereby 0.00 indicates symmetrical performance, and +1.00 indicates detection of the rightmost item only (i.e., a very strong lateralized performance to the right side, indicating severe neglect).

Neglect severity	N+ patients	N+ with hemia nopia	N+ with quadrant anopia	N+ with VHFD	N+ without VHFD	CoC letters	CoC stars	CoC lines
Borderline	8	3	0	**3**	**5**	0.00 ± 0.01	-0.01 ± 0.01	0.01 ± 0.02
Mild	10	2	3	**5**	**5**	0.14 ± 0.09	0.10 ± 0.09	0.02 ± 0.03
Moderate	12	3	1	**4**	**8**	0.41 ± 0.21	0.38 ± 0.22	0.17 ± 0.25
Severe	14	8	1	**9**	**5**	0.85 ± 0.13	0.76 ± 0.21	0.34 ± 0.35

Supplementary Table S3. Sensitivity, specificity, PPV and NPV (incl. 95% CI) for the qualitative visual field assessment are reported for the **subsample of right-brain-damaged (RBD) stroke patients with neglect (n = 44)**. Results are reported for: a) individual four tests, and b) their combinations. Sensitivity, specificity, PPV, and NPV values are based on true/false positive/negative cases (cf. Supplementary Table S1) determined by comparing the qualitative visual field tests binary outcome (i.e., presence or absence of a deficit) with the SAP outcome. The best combination of two tests (see Discussion in the paper) is highlighted in bold text.

	**Stroke patients with RBD (only patients with neglect: n = 44)**			
**a) Individual tests**	**Sensitivity (%)**	**Specificity (%)**	**PPV (%)**	**NPV (%)**
Face description	9.5 (-3.0 – 22.1)	100 (100 – 100)	100 (100 – 100)	52.5 (37.4 – 67.6)
Binocular static finger wiggle	61.9 (41.1 – 82.7)	100 (100 – 100)	100 (100 – 100)	72.4 (56.7 – 88.1)
Monocular static finger wiggle	90.5 (77.9 – 100)	100 (100 – 100)	100 (100 – 100)	91.3 (80.3 – 100)
Kinetic boundary perimetry	90.5 (77.9 – 100)	100 (100 – 100)	100 (100 – 100)	91.3 (80.3 – 100)
**b) Combinations of tests**				
**2-test**				
Face description + Binocular static finger wiggle	61.9 (41.1 – 82.7)	100 (100 – 100)	100 (100 – 100)	72.4 (56.7 – 88.1)
Face description + Monocular static finger wiggle	90.5 (77.9 – 100)	100 (100 – 100)	100 (100 – 100)	91.3 (80.3 – 100)
Binocular static finger wiggle + Monocular static finger wiggle	90.5 (77.9 – 100)	100 (100 – 100)	100 (100 – 100)	91.3 (80.3 – 100)
Face description + Kinetic boundary perimetry	95.2 (86.1 – 100)	100 (100 – 100)	100 (100 – 100)	95.5 (87.1 – 100)
Binocular static finger wiggle + Kinetic boundary perimetry	100 (100 – 100)	100 (100 – 100)	100 (100 – 100)	100 (100 – 100)
Monocular static finger wiggle + Kinetic boundary perimetry	**100 (100 – 100)**	**100 (100 – 100)**	**100 (100 – 100)**	**100 (100 – 100)**
**3-test**				
Face description + Binocular static finger wiggle + Monocular static finger wiggle	90.5 (77.9 – 100)	100 (100 – 100)	100 (100 – 100)	91.3 (80.3 – 100)
Face description + Binocular static finger wiggle + Kinetic boundary perimetry	100 (100 – 100)	100 (100 – 100)	100 (100 – 100)	100 (100 – 100)
Face description + Monocular static finger wiggle + Kinetic boundary perimetry	100 (100 – 100)	100 (100 – 100)	100 (100 – 100)	100 (100 – 100)
Binocular static finger wiggle + Monocular static finger wiggle + Kinetic boundary perimetry	100 (100 – 100)	100 (100 – 100)	100 (100 – 100)	100 (100 – 100)
**4 -test**				
Face description + Binocular static finger wiggle + Monocular static finger wiggle + Kinetic boundary perimetry	100 (100 – 100)	100 (100 – 100)	100 (100 – 100)	100 (100 – 100)
